# Validation of the motion sickness severity scale: Secondary analysis of a randomized, double-blind, placebo-controlled study of a treatment for motion sickness

**DOI:** 10.1371/journal.pone.0280058

**Published:** 2023-01-05

**Authors:** Mark É. Czeisler, Justina M. Pruski, Pan Wang, Jingyuan Wang, Changfu Xiao, Mihael H. Polymeropoulos, Vasilios M. Polymeropoulos

**Affiliations:** Vanda Pharmaceuticals, Washington, DC, United States of America; UNITED STATES

## Abstract

**Background:**

Motion sickness is characterized by nausea and vomiting among a constellation of symptoms. Symptom severity is dynamic and distressing. Most validated motion sickness scales are time-intensive and effortful, with alternative scales having uncertain performance or non-specific measures. A validated instrument allowing for facile, rapid assessment of core motion sickness symptom severity would therefore be valuable. We assessed the performance of the Motion Sickness Severity Scale (MSSS), a six-item questionnaire designed to measure real-time motion sickness symptoms.

**Methods:**

MSSS construct validity was assessed as a secondary analysis of data from 63 healthy participants without antiemetic treatment in a clinical trial (Unique Identifier = NCT03772340) conducted to evaluate the safety and efficacy of Tradipitant—a novel neurokinin-1 receptor antagonist—in the treatment of motion sickness. Clinical outcome assessments included the MSSS, the Patient Global Impression of Severity (PGI-S), and the Motion Sickness Assessment Questionnaire (MSAQ). The performance of the MSSS through Pearson correlation coefficients, within-group analysis of variance, empirical cumulative distribution functions, and Kolmogorov-Smirnov tests.

**Results:**

The MSSS correlated very highly with the PGI-S (r = 0.93, p-value<0.0001) and highly with the MSAQ (r = 0.83, p-value<0.0001). Mean MSSS scores between increasing PGI-S severity levels increased significantly in all four increments (None-to-Mild: p-value = 0.006, Mild-to-Moderate: p-value<0.0001, Moderate-to-Severe: p-value = 0.006, Severe-to-Very-Severe: p-value = 0.002). There were statistically significant differences in MSSS score distributions stratified by PGI-S severity level, with higher MSSS scores associated with higher PGI-S severity levels and lower MSSS scores associated with lower PGI-S severity levels.

**Discussion:**

The MSSS is a valid instrument for the assessment of the core motion sickness symptoms and is reflective of global disease severity. Implementation of the MSSS and comparable simplified, short questionnaires in motion sickness research will provide rapid and accurate measures of disease severity. These measures will enable further elucidation of motion sickness as an illness and inform the development and evaluation of motion sickness therapies.

## Introduction

Several questionnaires have been developed to assess motion sickness [1], which affects up to 30% of the U.S. population [2]. Many such questionnaires, including The Nausea Profile (NP) [3], which provides a detailed assessment of the nausea symptom of motion sickness, the Motion Sickness Assessment Questionnaire (MSAQ) [4], which provides a detailed assessment of the constellation of motion sickness symptoms, and the Simulator Sickness Questionnaire (SSQ) [5], developed to assess simulator sickness (i.e., motion sickness in the absence of actual motion, [6]), have been widely used to assess motion sickness [1]. However, fewer questionnaires have been developed to assess motion sickness during stimulus presentation, commonly owing to the length of the questionnaire, the distraction of the main task, and the impracticality of completing questionnaires while experiencing unpleasant motion sickness symptoms [7]. To our knowledge, the only such measure evaluated before our study was the Fast MS Scale (FMS), a verbal rating scale developed by Keshavarz and Hecht in 2011 in which respondents are instructed to complete a 20-point scale verbally once per minute during motion stimulus. While the FMS provides rapid and easy-to-complete measures of motion sickness symptoms, it may be challenging to administer at scale, such as during a clinical trial with many participants exposed to a stimulus simultaneously. It would therefore be useful to have a valid scalable, rapid, and easy-to-complete self-report measure of motion sickness symptom severity.

The Motion Sickness Severity Scale (MSSS), previously used in an efficacy study of Hyoscine (Scopolamine) [8], would meet this need, as it allows for the repeated and rapid assessment of motion sickness symptoms with emphasis on its core symptoms of nausea and vomiting [9]. There is high interindividual variability in additional symptoms associated with motion sickness, which include but are not limited to drowsiness, pallor, cold sweating, increased salivation, headache, and irritability [10]. The MSSS was utilized in the Motion Sifnos clinical trial (ClinicalTrials.gov Identifier NCT03772340), a study to investigate the efficacy of Tradipitant for individuals affected by motion sickness, because the stimulating conditions of the study and degree of impairment associated with nausea and vomiting demanded an assessment that could provide rapid and informative measures of illness without overly burdening participants. The MSSS is a 7-point scale rated from 0–6 on which respondents select the item that best describes their current state: none (0), stomach awareness or discomfort (1), mild nausea (2), moderate nausea (3), severe nausea (4), retching (5), or vomiting (6). The scale increases according to the established progression of motion sickness symptomatology, with a focus on the core symptoms [9, 11]. While the MSSS was constructed with sound theoretical development, it has not yet been robustly validated against established motion sickness measures.

Similarly, the 11-point Motion Illness Symptoms Classification (MISC) scale, which was also developed to assess motion sickness symptomatology during stimulus presentation, has been employed prior to its performance evaluation [12]. Encouragingly, and underscoring the need for instruments such as the MSSS, after the Motion Sifnos clinical trial was conducted, Reuten *et al*. published a study that included assessment of the MISC [13]. Using data from 216 participants pooled from seven different experiments, the authors reported that motion sickness symptoms manifest in a fixed order, while unpleasantness progresses non-monotonically, indicating that measuring symptomatology would be less ambiguous than measuring unpleasantness in the assessment of motion sickness severity. This evaluation of the MISC offered an opportunity to evaluate the MSSS and discuss the similarities and differences between these scales.

Therefore, in this secondary analysis of data from a clinical efficacy study (Motion Sifnos) for the development of Tradipitant (a novel neurokinin-1 antagonist) for the treatment of motion sickness [14], we sought to assess the validity of the MSSS compared with the Patient Global Impression of Severity (PGI-S), a brief measure of symptom severity, and the MSAQ, a validated motion sickness scale. The PGI-S is a single-item, 5-point scale that evaluates the global severity of a given sickness or disease [15–17]. The MSAQ is a 16-item, 9-point questionnaire that evaluates motion sickness as a multidimensional construct and includes many of the variably experienced symptoms classified into the following factors: gastrointestinal, central, peripheral, and sopite-related. If valid, the MSSS would provide clinicians and researchers with a rapid, easy-to-administer, and easy-to-complete measure of motion sickness severity designed for administration during stimulus presentation.

## Materials and methods

This manuscript reports finding from a secondary analysis of data collected in the Motion Sifnos Study [14]. Construct validity of the MSSS was evaluated by analyzing the relationship between scores on the MSSS and both the PGI-S and MSAQ from the placebo group in the Motion Sifnos Study, comprised of healthy individuals without antiemetic treatment.

### Motion Sifnos Study

#### Participants

A total of 126 participants (97 female, 29 male, median age: 36.5 years) were recruited from the greater Los Angeles, California, USA area through radio and online streaming advertisements. Participants completed the Motion Sickness Eligibility Questionnaire (MSEQ), a phone interview, and an in-person medical screening. Screening for participation criteria included medical and psychiatric history, physical examination, electrocardiography, serum chemistry, hematology, urinalysis, and urine toxicology. Participants who met the inclusion criteria had a significant history of motion sickness, were otherwise in good health, and had no history of any other nausea-inducing disorders. The institutional review board at Advarra (Columbia, Maryland, USA) reviewed and approved the study protocol and provided ethical oversight for the study. All participants provided written informed consent prior to enrollment, and all methods were carried out in accordance with relevant guidelines and regulations.

#### Instruments

**Fig 1:**
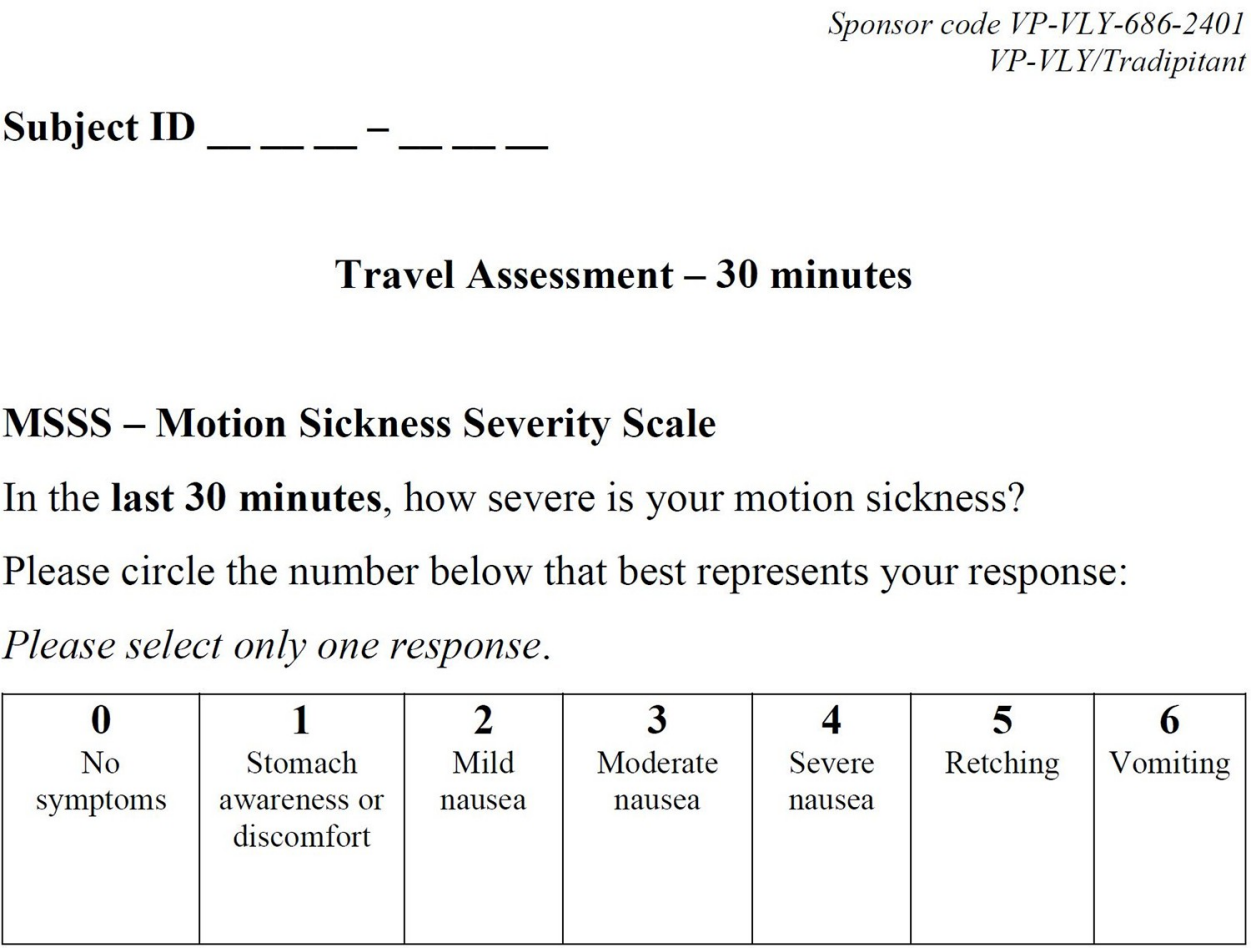
The Motion Sickness Severity Scale (MSSS). Participants completed the MSSS every 30 minutes of the boat trip.

Motion Sickness Severity Scale (MSSS)–The MSSS is a self-reported clinical outcome assessment that evaluates the severity of motion sickness symptoms, including the core symptoms nausea and vomiting. In 30-minute intervals throughout the duration of the boat trip, participants rated the severity of their motion sickness symptoms on a 7-point scale: no symptoms, stomach awareness or discomfort, mild nausea, moderate nausea, severe nausea, retching, or vomiting (Fig 1).

**Fig 2:**
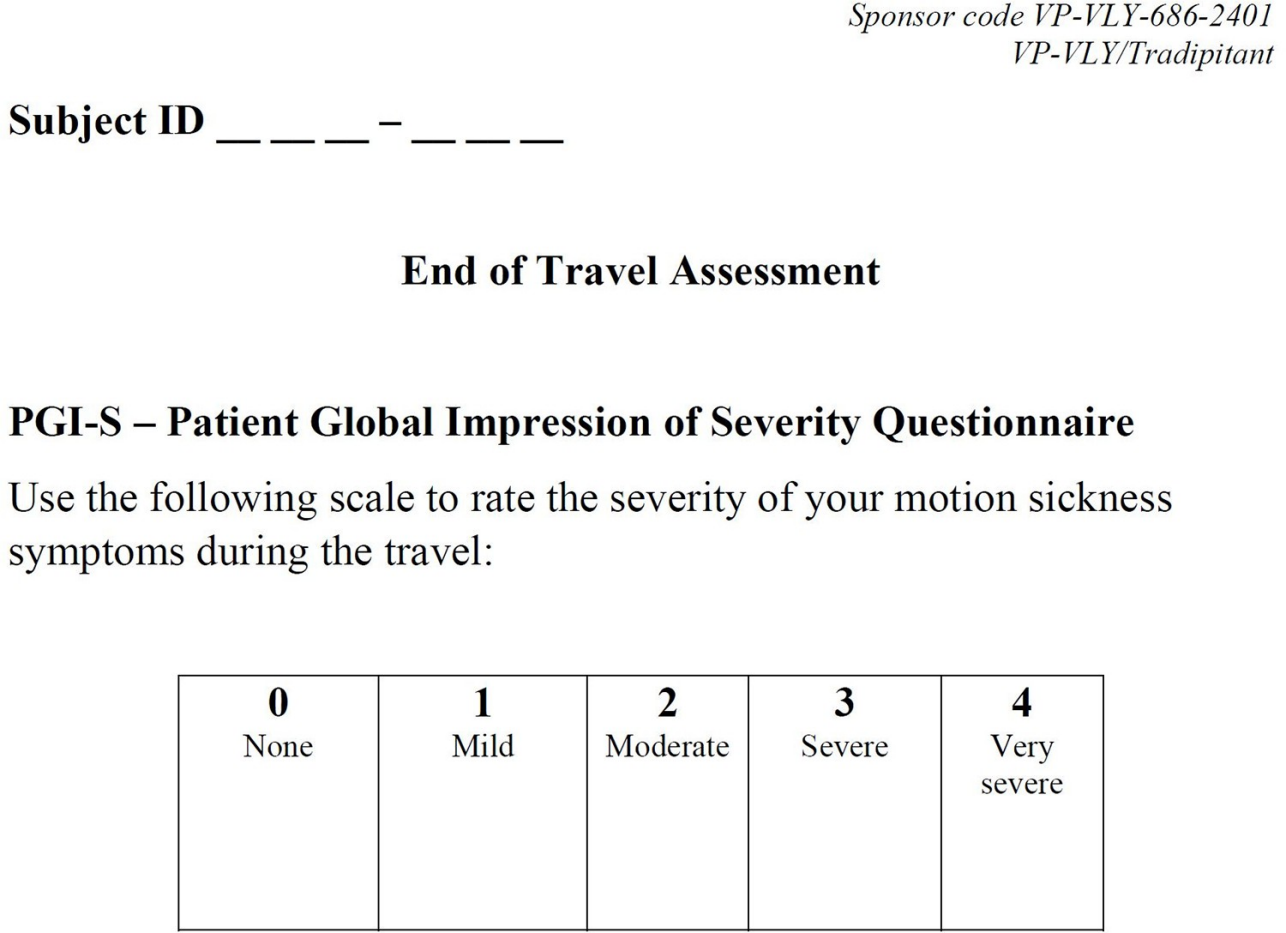
The Patient Global Impression of Severity scale (PGI-S). Participants completed the PGI-S approximately 60 minutes after the conclusion of the boat trip.

Patient Global Impression of Severity (PGI-S)–The PGI-S is a self-reported clinical outcome assessment that evaluates the global severity of a sickness or disease. Approximately 60 minutes following the boat trip, participants rated the severity of their motion sickness on a 5-point scale: none, mild, moderate, severe, or very severe (Fig 2).

**Fig 3:**
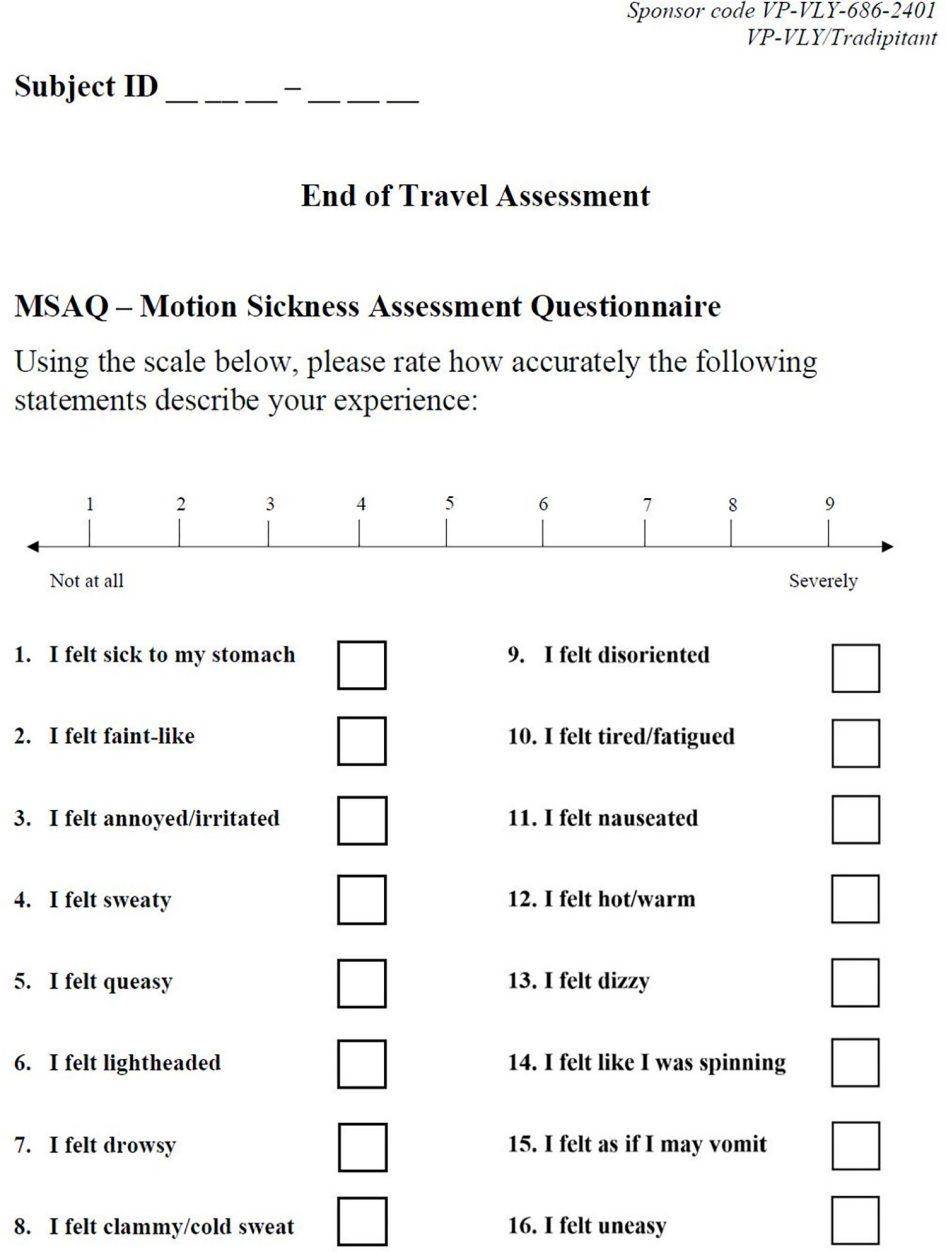
The Motion Sickness Assessment Questionnaire (MSAQ). Participants completed the MSAQ approximately 60 minutes after the conclusion of the boat trip.

Motion Sickness Assessment Questionnaire (MSAQ)–The MSAQ is a self-reported clinical outcome assessment that evaluates the severity of motion sickness with specificity for individual symptoms that may be experienced. Approximately 60 minutes following boat travel, participants rated their degree of agreement with each of 16 items (statements) about different motion sickness symptoms on a 9-point scale from “Not at all” to “Severely” (Fig 3). The MSAQ Total score is calculated by summing the average of all 16 items and converting the score to a percent. Items are categorized into four groups (Gastrointestinal, Central, Peripheral, and Sopite-related) to distinguish between the dysregulated systems affected by motion sickness. Scores for these groups are also calculated by converting sum of the items in the factor and converting the raw score to a percent. Item 11 “I felt nauseated” (MSAQ Nausea) and Item 15 “I felt as if I may vomit” (MSAQ Vomit) evaluate the severity of nausea and vomiting. In addition to the total MSAQ score, the four factors MSAQ-GI, MSAQ-C, MSAQ-P, and MSAQ-SR, and MSAQ Nausea and MSAQ Vomit, were independently evaluated and referred to as MSAQ subscales.

#### Procedure

Eligible participants were randomized to take Tradipitant 170mg or placebo approximately 60 minutes prior to the initiation of boat travel. Groups of between 10 and 26 participants each participated in one of seven boat trips lasting between 237 and 250 minutes, except where extreme sea conditions limited one trip (Boat 5) to 148 minutes. Boat travel occurred in the Pacific Ocean near Los Angeles between January and May 2019. Participants rated their motion sickness symptom severity using the MSSS in 30-minute intervals throughout the trip. Participants also completed the PGI-S and MSAQ approximately 60 minutes after the conclusion of the trip. Additional information about the study procedure is available in the study efficacy report [14].

### Evaluation of the construct validity of the MSSS

#### Participant groups

To eliminate treatment effects of neurokinin-1 receptor antagonism (which has demonstrated anti-nauseogenic and antiemetic properties) as a potential confounder of validity testing, the Tradipitant group was excluded from the primary evaluation of the validity of the MSSS. The decision to conduct the primary evaluation using data from healthy participants without antiemetic treatment is supported by a demonstrated treatment effect, including significantly reduced vomiting observed in the Tradipitant group compared to placebo (Tradipitant incidence of vomiting = 17.5%, placebo incidence of vomiting = 39.7%, difference = 22.2%, p-value = 0.0039 [Cochran-Mantel-Haenszel test adjusting for boat trip]) [14]. As an exploratory analysis further described below, Pearson correlation coefficients between the MSSS and both the PGI-S and MSAQ were calculated for the overall Motion Sifnos Study population, and for the Tradipitant treatment group. These tests provide insight as to how the inclusion of the interventional group and any associated treatment effects may have influenced the relationships between questionnaire scores.

#### Statistical analyses

Statistical analyses performed included Pearson correlations, one-way analysis of variance (ANOVA), empirical cumulative distribution functions (ECDF), and two-sample Kolmogorov–Smirnov tests (K-S test). To evaluate the relationship between mean scores across questionnaires, analyses were anchored to the global PGI-S and compared to the motion-sickness-specific questionnaires (MSSS and MSAQ).

There were four core elements of the validation study. Within the analytic sample (placebo group, n = 62), first, Pearson correlation coefficients were calculated to estimate the linear association between MSSS scores and each of PGI-S and MSAQ scores. Second, in subpopulations stratified by the five PGI-S severity levels (none, mild, moderate, severe, very severe), within-group differences in mean MSSS and MSAQ scores were evaluated using a one-way ANOVA [18]. Third, the distributions of MSSS and MSAQ scores corresponding to each PGI-S severity were evaluated through ECDFs. Fourth, and the equality of the ECDF was assessed via a K-S test [19].

There were two additional exploratory analyses of the validation study. First, we evaluated the Pearson correlation between the MSSS and subscales of the MSAQ, including items conceptually directly related to MSSS scores (i.e., the vomit [item 11] and nausea [item 15] MSAQ items) and the group of gastrointestinal MSAQ items. Second, we calculated Pearson’s correlation of MSSS and PGI-S and MSAQ scores on the overall Motion Sifnos Study population (treatment and placebo groups, n = 125). The purpose of this analysis was to evaluate whether correlations differed with the expanded study population that included a group on an investigational antiemetic therapeutic.

The significance level of all tests was set at α = 0.05 given that the statistical tests were performed for different purposes and nonduplicative. Multiple statistical measures were used without thresholds to characterize the performance of the MSSS relative to established measures.

Pearson’s correlation coefficient is a measurement of association between two variables. The strength of association between variables is described by -1.0≤r≤+1.0 where -1.0 represents a perfect negative correlation and +1.0 represents a perfect positive correlation. Pearson’s correlation was chosen rather than Spearman’s correlation to assess the relationship between continuous rather than ranked scale scores. Using guidelines established for appropriate use of correlation for medical research, a threshold of r ≥│0.70│was used to interpret correlations as high (│0.70│≤r ≤│0.90│) or very high (r>│0.90│) [20].

A one-way analysis of variance (ANOVA) was used to compare MSSS (or MSAQ) in subgroups corresponding to consecutive and ascending PGI-S severity levels. Statistically significant increases in mean scores accompanying ascending PGI-S levels would be indicative of an association between the MSSS (or MSAQ) and the validated questionnaires.

An empirical cumulative distribution function (ECDF) of a sample is an ascending function ranging from 0 to 1 over all possible values. At each point *x*, the ECDF value at *x* is the percentage of observations that are less or equal to *x*. ECDFs of MSSS scores were generated for subpopulations corresponding to each PGI-S severity level. When comparing ECDFs, increasingly right ECDFs correspond to stochastically greater values. Non-parametric kernel smoothing was applied to ECDF curves.

The Kolmogorov-Smirnov test (K-S test) serves as a nonparametric test to compare two samples. K-S tests were applied to MSSS scores corresponding to increasing PGI-S severity levels. The underlying hypothesis of the K-S test is that the two ECDFs were generated from the same population-level Cumulative Distribution Function (CDF). Statistically significant K-S test results between MSSS score ECDFs at increasing PGI-S severity levels would indicate that MSSS scores between PGI-S severity levels were likely sampled from different distributions and that MSSS scores are differentiated by PGI-S severity level.

## Results

**Fig 4:**
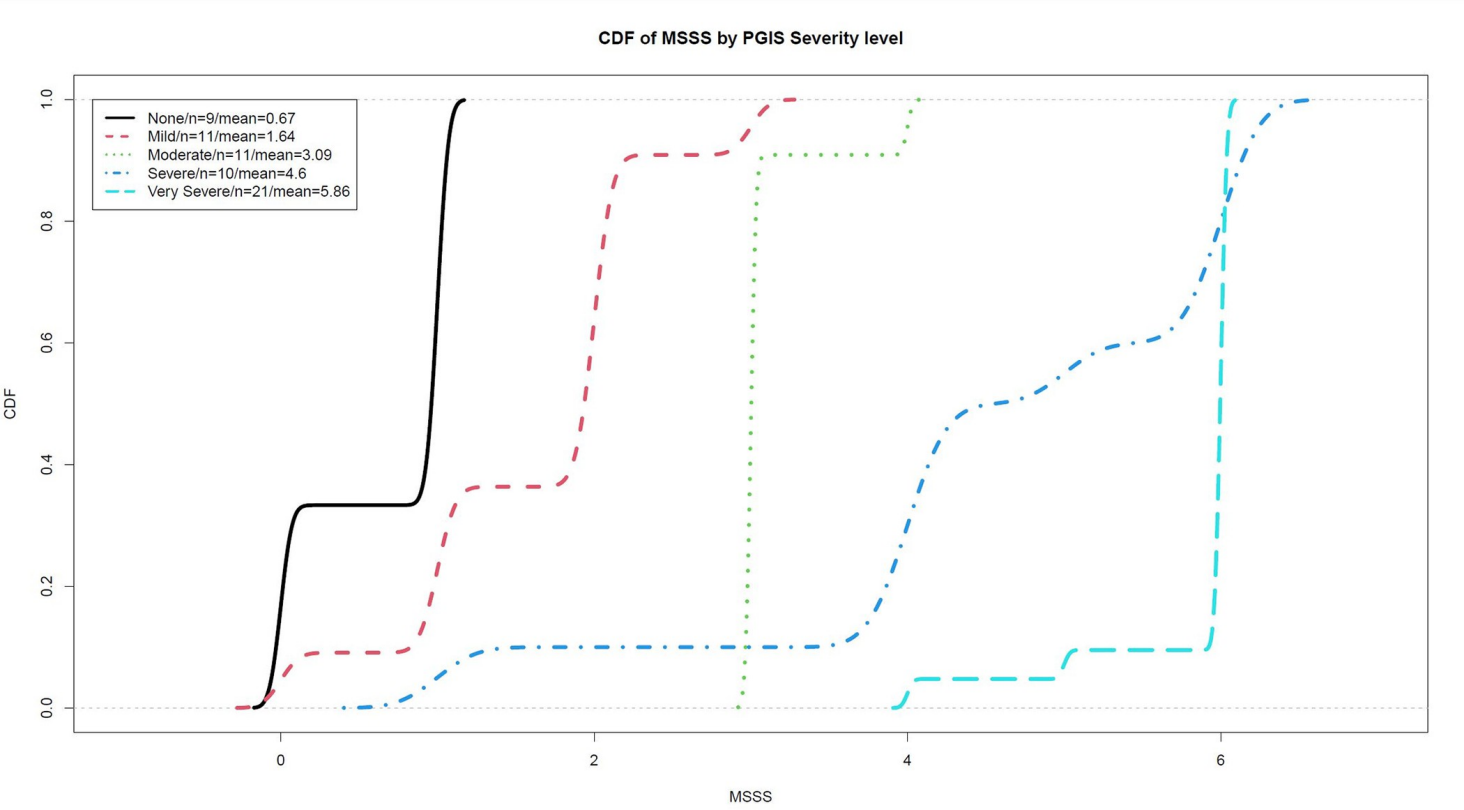
Empirical Cumulative Distribution Functions (ECDFs) of MSSS scores plotted for each PGI-S severity level. The empirical cumulative distribution functions of MSSS scores recorded by participants in each PGI-S severity level are plotted, with non-parametric kernel smoothing applied to produce curves. The order and separation of PGI-S-score-lines indicate that MSSS score distributions include greater proportions of high scores with increasing PGI-S severity level.

Included in the analysis were 62 healthy participants without antiemetic treatment who completed the PGI-S, MSAQ, and MSSS per the study protocol. Paired questionnaire scores of the MSSS and the PGI-S, MSAQ, and MSAQ-GI from individual participants are presented in scatterplots as panels A, C, and D of Fig 4. Mean questionnaire scores of participants grouped by PGI-S severity level are reported in Table 1. In addition to the mean MSAQ total score, mean scores for the MSAQ-GI, MSAQ Nausea, and MSAQ Vomit are reported.

**Table 1:** Mean MSSS, MSAQ and MSAQ subscale scores grouped by PGI-S severity level. Mean questionnaire scores grouped by PGI-S severity level are reported for healthy participants without antiemetic treatment in the Motion Sifnos Study. Mean scores increase incrementally across the MSSS, MSAQ, and all subscales with increasing PGI-S severity levels.

PGI-S Severity Level	N^1^ (% of N^2^)	Mean MSSS Worst Score (0–6) (SD^3^)	Mean MSAQ Total Score (%) (SD^3^)	Mean MSAQ Item 11: Nausea Score (1–9) (SD^3^)	Mean MSAQ Item 15: Vomiting Score (1–9) (SD^3^)	Mean MSAQ-GI Factor Score (%) (SD^3^)
**0–None**	9 (14.5)	0.67 (0.5)	16.13 (1.2)	1.00 (0)	1.00 (0)	11.42 (0.9)
**1–Mild**	11 (17.7)	1.64 (0.8)	23.74 (8.5)	2.27 (1.5)	1.82 (1.6)	22.98 (15.3)
**2–Moderate**	11 (17.7)	3.09 (0.3)	42.17 (9.7)	5.27 (1.7)	4.64 (2.4)	53.79 (16.9)
**3–Severe**	10 (16.1)	4.60 (1.6)	63.61 (21.4)	7.60 (1.8)	7.90 (2.2)	80.28 (20.7)
**4–Very Severe**	21 (33.9)	5.86 (0.5)	72.09 (19.1)	8.71 (1.0)	8.86 (0.5)	95.90 (8.6)
**Total**	62 (100)	3.66 (2.1)	48.71 (26.7)	5.66 (3.3)	5.56 (3.5)	60.71 (35.9)

1. number of participants in PGI-S severity level

2. total participants without antiemetic treatment included in the analysis (n = 62)

3. standard deviation

MSSS scores correlated very highly with scores on the PGI-S (r = 0.93, p-value<0.0001) (Table 2), and highly or very highly on the MSAQ, MSAQ-GI, MSAQ Vomit, and MSAQ Nausea (MSAQ: r = 0.83, MSAQ-GI: r = 0.92, MSAQ Nausea: r = 0.91, MSAQ Vomit: r = 0.90, all p-values<0.0001) (Table 3).

**Table 2:** Pearson correlations between PGI-S scores and other questionnaire scores. Pearson correlations between PGI-S scores and scores reported on the other questionnaires are high or very high across all assessment instruments. For all Pearson correlation coefficients, p-value<0.0001.

Treatment Group	MSSS Worst Score	MSAQ Total Score	MSAQ Item 11: Nausea Score	MSAQ Item 15: Vomiting Score	MSAQ-GI Factor Score
**Placebo**	0.93	0.83	0.91	0.90	0.92
**Overall**	0.88	0.83	0.86	0.86	0.90
**Tradipitant**	0.79	0.84	0.79	0.83	0.86

**Table 3:** Pearson correlations between MSAQ individual items, factors, and other questionnaires. Pearson correlation coefficients between individual and grouped MSAQ items and the other questionnaires are presented in the table for the overall Motion Sifnos Study population, as well as for the placebo and Tradipitant treatment groups. High (r≥0.70) and very high (r>0.90) correlations are bolded.

	*Overall*			*Placebo*			*Tradipitant*		
MSAQ Item(s)	MSSS Worst Score	PGI-S Severity Level	MSAQ Total Score	MSSS Worst Score	PGI-S Severity Level	MSAQ Total Score	MSSS Worst Score	PGI-S Severity Level	MSAQ Total Score
**Item 1**	**0.78** ^*^	**0.83** ^*^	**0.82** ^*^	**0.85** ^*^	**0.86** ^*^	**0.81** ^*^	**0.70** ^*^	**0.81** ^*^	**0.84** ^*^
Sick to Stomach									
**Item 2**	0.53	0.61	**0.81** ^*^	0.53	0.56	**0.77** ^*^	0.54	0.69	**0.86** ^*^
Faint-like									
**Item 3**	0.56	0.56	**0.73** ^*^	0.61	0.55	**0.72** ^*^	0.47	0.56	**0.73** ^*^
Annoyed/Irritated									
**Item 4**	0.56	0.64	**0.77** ^*^	0.59	0.62	**0.74** ^*^	0.53	0.68	**0.81** ^*^
Sweaty									
**Item 5**	**0.80** ^*^	**0.83** ^*^	**0.85** ^*^	**0.88** ^*^	**0.89** ^*^	**0.85** ^*^	0.68	**0.76** ^*^	**0.85** ^*^
Queasy									
**Item 6**	0.58	0.61	**0.86** ^*^	0.67	0.63	**0.87** ^*^	0.49	0.60	**0.84** ^*^
Lightheaded									
**Item 7**	0.29	0.25	0.50	0.32	0.21	0.47	0.30	0.33	0.55
Drowsy									
**Item 8**	0.60	0.65	**0.80** ^*^	0.62	0.61	**0.79** ^*^	0.58	**0.70** ^*^	**0.81** ^*^
Clammy/Cold Sweat									
**Item 9**	0.50	0.55	**0.79** ^*^	0.55	0.52	**0.80** ^*^	0.45	0.60	**0.79** ^*^
Disoriented									
**Item 10**	0.49	0.43	0.65	0.55	0.49	**0.71** ^*^	0.43	0.37	0.60
Tired/Fatigued									
**Item 11**	**0.85** *	**0.86** *	**0.84** *	**0.93** **	**0.91** **	**0.86** *	**0.75** *	**0.79** *	**0.83** *
Nauseated									
**Item 12**	0.55	0.53	**0.74** *	0.55	0.54	**0.79** *	0.53	0.50	0.68
Hot/Warm									
**Item 13**	0.62	0.62	**0.83** *	0.64	0.58	**0.84** *	0.61	0.69	**0.82** *
Dizzy									
**Item 14**	0.60	0.63	**0.80** *	0.69	0.63	**0.86** *	0.46	0.63	**0.72** *
Spinning									
**Item 15**	**0.85** *	**0.86** *	**0.82** *	**0.92** * *	**0.90** *	**0.83** *	**0.76** *	**0.83** *	**0.81** *
May Vomit									
**Item 16**	**0.74** *	**0.76** *	**0.83** *	**0.83** *	**0.76** *	**0.84** *	0.62	**0.76** *	**0.83** *
Uneasy									
**Gastrointestinal Factor**	**0.87** *	**0.90** *	**0.88** *	**0.93** **	**0.92** **	**0.87** *	**0.78** *	**0.86** *	**0.90** ^**^
**Central Factor**	0.64	0.68	0.92**	0.69	0.66	**0.93** ^**^	0.58	**0.73** *	**0.92** *
**Peripheral Factor**	0.63	0.67	**0.85** *	0.64	0.64	**0.84** *	0.61	**0.70** *	**0.86** *
**Sopite-Related Factor**	0.67	0.65	**0.87** *	**0.75** *	0.66	**0.88** *	0.58	0.65	**0.86** *
**MSAQ Total**	**0.80** *	**0.83** *	**1.00** **	**0.86** *	**0.83** *	**1.00** **	**0.73** *	**0.84** *	**1.00** **

* denotes high correlation (r≥0.70)

** denotes very high correlation (r>0.90)

**Fig 5:**
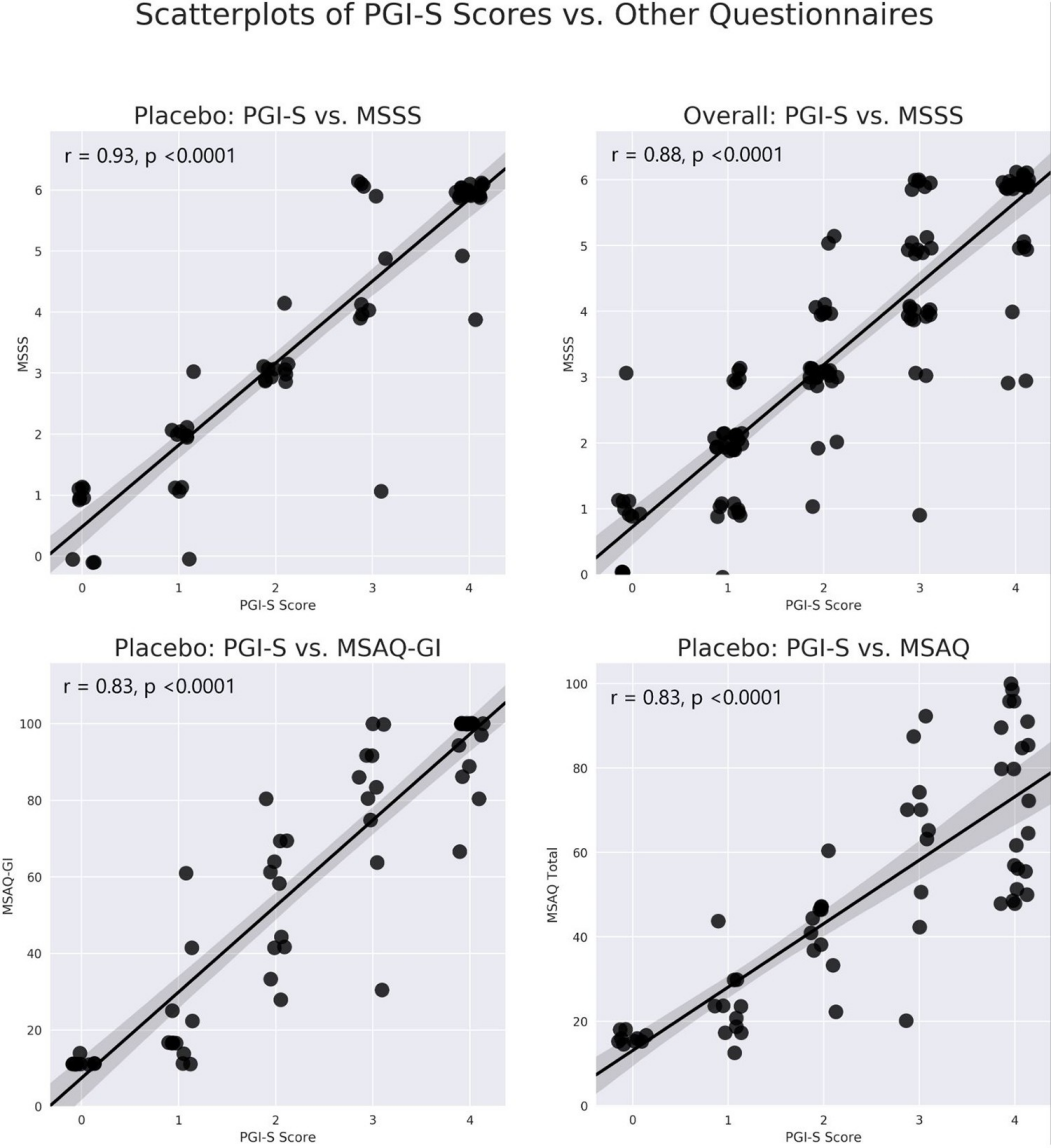
Scatterplots of MSSS scores paired to other questionnaire scores. The top two panels present scatterplots of individual participants scores on the PGI-S and MSSS in the placebo group (A) and overall Motion Sifnos population (B). Scatterplots of individual participants scores on the PGI-S and MSAQ-GI (C) and MSAQ Total (D) are shown. A linear regression line with a 95% confidence interval (the shaded area) is superimposed on each scatterplot. The scatterplots of PGI-S scores and the corresponding scores on each of the other assessment instruments reveal the relationship and distribution of paired scores, which is strong in all four panels.

MSSS scores increased significantly with increasing PGI-S severity levels across all four increments (Table 4). For example, the mean MSSS worst score was 0.97 points (out of 6) higher for patients whose PGI-S severity was mild (1 out of 4) as compared to none (0 out of 4) (p-value = 0.006). MSAQ and subscale scores also increased significantly with increasing PGI-S severity levels for majority of the comparisons (11/16, 68.75%). The ECDFs of MSSS are shifted to the right by ascending PGI-S severity, which indicate that higher PGI-S severity levels are associated with higher MSSS scores and lower PGI-S severity levels are associated with lower MSSS scores (Fig 5). Additionally, K-S test comparing MSSS scores corresponding to consecutive ascending PGI-S severity levels are all statistically significant.

**Table 4:** Differences in MSSS, MSAQ, and MSAQ subscale scores by PGI-S severity level. Least-squared (LS) means differences and p-values are reported from one-way analyses of variance with the main effect of PGI-S conducted to evaluate differences in mean scores on the MSSS, MSAQ, and subscales for healthy participants without antiemetic treatment.

Within-Group ANOVA					
Adjacent PGI-S Severity Levels	Mean [95% CI] MSSS Worst Score Least Squared Means Difference (p-value)	Mean [95% CI] MSAQ Total Score Least Squared Means Difference (p-value)	Mean [95% CI] MSAQ Item 11: Nausea Score Least Squared Means Difference (p-value)	Mean [95% CI] MSAQ Item 15: Vomiting Score Least Squared Means Difference (p-value)	Mean [95% CI] MSAQ-GI Factor Score Least Squared Means Difference (p-value)
**0 –None**	**0.97 [0.3, 1.6]**	**7.61 [1.6–13.6]**	**1.27 [0.2–2.3]**	0.82 [-0.3–1.9]	**11.56 [0.8–22.3]**
**1 –Mild**	**(0.006)**	**(0.02)**	**(0.02)**	(0.53)	**(0.04)**
**1 –Mild**	**1.45 [0.9–2.0]**	**18.43 [10.3–26.6]**	**3.00 [1.6–4.4]**	**2.82 [1.0–4.6]**	**30.81 [16.5–45.1]**
**2 –Moderate**	**(<0.0001)**	**(0.001)**	**(0.0003)**	**(0.004)**	**(0.0002)**
**2 –Moderate**	**1.51 [0.5–2.5]**	**21.44 [6.5–36.3]**	**2.33 [0.7–3.9]**	**3.26 [1.2–5.4]**	**26.49 [9.3–43.7]**
**3 –Severe**	**(0.006)**	**(0.007)**	**(0.0061)**	**(0.004)**	**(0.004)**
**3 –Severe**	**1.26 [0.5–2.0]**	8.48 [-7.1–24.5]	**1.11 [0.1–2.1]**	0.96 [0.0–2.0]	**15.62 [5.0–26.3]**
**4 –Very Severe**	**(0.002)**	**(**0.27**)**	**(0.03)**	**(**0.49**)**	**(0.006)**

In the exploratory analysis of the overall Motion Sifnos Study population (treatment and placebo groups), the correlation remained high between the MSSS and the PGI-S (r = 0.88, p-value<0.0001) (Table 2), as well as between the MSSS and the MSAQ and MSAQ-GI factor (MSAQ: r = 0.80, p-value<0.0001, MSAQ-GI: r = 0.87, p-value<0.0001) (Table 3). Paired questionnaire scores of the MSSS and PGI-S for the overall population are presented in panel B of Fig 4. For Pearson correlations between the MSSS individual and grouped MSAQ items, across all three groups (overall, placebo, treatment) five MSAQ items (1, 5, 11, 15, 16) correlated highly or very highly with at least 8/9 (89%) of the other questionnaires. Four of these items make up the MSAQ-Gastrointestinal Factor (Items 1, 5, 11, 15). None of the other eleven MSAQ items correlated highly in more than 50% of the other questionnaires. Accordingly, the other factors (Central, Peripheral, and Sopite-Related) have relatively lower correlations to the other questionnaires (0.58≤r≤0.75) but correlate highly to the total MSAQ score (0.84≤r≤0.93).

## Discussion

Evaluation of the construct validity of the MSSS was motivated by the desire for a reliable diagnostic instrument to assess the severity of motion sickness with the following elements: measurement of core symptoms, brevity, clarity of scale items for undemanding completion, potential for repeated and rapid assessment of acute motion sickness severity during stimulus presentation (including when the respondent is distressed), and scalability. To evaluate the MSSS with attention to these elements, the scale was compared to two validated assessments: a brief questionnaire (PGI-S) used as an index of global illness severity, and a comprehensive questionnaire (MSAQ) used as an assessment of the multiple dimensions of motion sickness. This analysis of the construct validity of the MSSS provides evidence that the MSSS correlates strongly with validated and widely used questionnaires and is therefore a valid instrument for the assessment of motion sickness.

Scores on the MSSS correlated very highly with scores on the PGI-S, and sufficient differentiation is supported by the within-group ANOVA demonstrating significant increases in mean MSSS scores with increasing PGI-S severity level. The order and shape of the PGI-S ECDFs reveal that grouped MSSS score distributions indicate that higher PGI-S severity levels are associated with higher MSSS scores, and lower PGI-S severity levels are associated with lower MSSS scores. This analysis suggests that the MSSS accurately measures motion sickness severity progression from no symptoms to stomach awareness and eventually to severe nausea and vomiting [2, 10]. These findings provide evidence that as a single-item questionnaire with comparable length to the PGI-S, the MSSS can assess the severity of motion sickness with specificity for its core symptoms.

Similarly, scores on the MSSS correlated highly with scores on the MSAQ. The high degree of association of scores between the questionnaires supports the construct validity of the MSSS based on the MSAQ, which was designed to capture the highly variable symptom profiles of motion sickness and validated against two widely used motion sickness severity assessment instruments: the Pensacola Diagnostic Index and Nausea Profile [4]. Scores from the MSAQ gastrointestinal factor correlated particularly highly with MSSS scores, suggesting that this factor was the primary driving force of the high correlation, while the other factors (central, peripheral, sopite-related) had more variable presentation among participants. This finding is in agreement with survey data collected to inform the development of the MSAQ in which an exploratory analysis found that gastrointestinal symptom descriptors accounted for 38% of the variance of symptom ratings (symptoms in the other three categories accounted for 17% combined) [4]. Given the high association between MSSS and the MSAQ gastrointestinal factor, it is likely that the MSSS captures the symptoms that have the greatest influence on global motion sickness severity. Correlations from the other factors are still significant but lower in magnitude, suggesting that their presentation still contributes to severity, but may do so in a less predictive manner given the variability by which they are experienced.

Together, the findings of this paper in evaluating the 7-item MSSS and those of Reuten *et al*. in evaluating the 11-item MISC suggest that with simplified and shorter questionnaires than the MSAQ, the MSSS and MISC assess motion sickness severity and reflect global disease state by focusing on core symptoms. This distinction highlights differences in purpose for questionnaires. The MSAQ can capture the multiple dimensions of motion sickness symptoms that may be experienced. The MSSS can rapidly and acutely measure severity of the core symptoms of motion sickness in a single-item questionnaire. The MISC provides yet another viable scale to assess motion sickness symptom severity, with enhanced resolution during pre-nauseated states [13]. Future research including both measures could explore similarities in and differences between these simplified symptom screening scales. Moreover, because each item on the MSAQ is weighted equally, each item within the MSAQ score is assessed by the intensity of each symptom regardless of the malaise associated with that symptom and the degree to which that symptom contributes to global burden of disease. The cumulative weight of high-intensity, low-malaise symptom scores (e.g., drowsiness, fatigue, body temperature) could increase total MSAQ scores in the absence of nausea or vomiting, just as low-intensity low-malaise scores could decrease total MSAQ scores when high-malaise symptoms such as severe nausea and vomiting are experienced.

Of note, the MSAQ was validated based on motion sickness ratings from 21 students following exposure to a rotating optokinetic drum [4]. While the MSAQ has been used widely since its development, the MSAQ scores collected in the Motion Sifnos Study—based on motion sickness ratings from exposure to sea travel—provide some of the first evidence supporting the MSAQ as a useful instrument for assessing the multiple dimensions of motion sickness symptoms provoked by real-world stimuli. Additionally, although the PGI-S is developed for broad application to measure symptom severity, it has not been validated to assess motion sickness symptoms specifically. Finally, the study inclusion criteria of a significant history of motion sickness may partially explain why females were overrepresented in this study, as females are more susceptible to motion sickness symptoms than are males, especially at younger ages [21]. Future research using the MSSS should evaluate the potential effects of gender, age, and motion sickness history on the validity of the MSSS. Additionally, the PGI-S and MSAQ were completed 60 minutes after the boat trip, subjecting these scores to marginal recall bias—future assessments of the MSSS could compare score estimates with other scales designed for administration during stimulus presentation.

This evaluation of the validity of the MSSS was performed on data collected in a single study in which participants with a history of motion sickness were exposed to different sea conditions and randomized to take Tradipitant or placebo as part of the Motion Sifnos Study, with the primary analysis focused on participants without antiemetic treatment. While these environmental and interventional variables may increase the variability in questionnaire scoring as potential confounders, results from the exploratory analysis further support the MSSS for assessing motion sickness. The robustness of scores with the inclusion of these variables indicates that the utility of the MSSS may extend across populations, interventions, and stimuli.

The findings reported in this study establish the MSSS as a highly performing instrument for the assessment of motion sickness designed for use during stimulus presentation, presenting motion sickness researchers with a valuable symptom-screening instrument comparable to the recently evaluated MISC. Further research using the MSSS for repeated assessment of motion sickness severity in larger populations with exposure to variable provocative stimuli will further inform its utility. Going forward, the MSSS can serve as a valuable instrument—in both clinical and research settings—that can be administered to assess motion sickness severity and reflect global state of disease.

## List of abbreviations

ANOVA: Analysis of Variance
CDF: Cumulative Distribution Function
ECDF: Empirical Cumulative Distribution Function
K-S Test: Kolmogorov–Smirnov test
LS: Least-squared
mg: Milligram(s)
MISC
MSAQ: Motion Sickness Assessment Questionnaire
MSAQ-GI: Motion Sickness Assessment Questionnaire Gastrointestinal Factor
MSEQ: Motion Sickness Eligibility Questionnaire
MSSS: Motion Sickness Severity Scale
PGI-S: Patient Global Impression of Severity
SD: Standard Deviation
